# Artificial Intelligence Based Approaches to Identify Molecular Determinants of Exceptional Health and Life Span-An Interdisciplinary Workshop at the National Institute on Aging

**DOI:** 10.3389/frai.2019.00012

**Published:** 2019-08-06

**Authors:** Jason H. Moore, Nalini Raghavachari, Workshop Speakers

**Affiliations:** ^1^University of Pennsylvania, Philadelphia, PA, United States; ^2^National Institute on Aging - NIH Bethesda, MD, United States

**Keywords:** health span and life span, GWAS, protective factors, systems approach, artificial intelligence, machine learning, deep learning, comparative biology

## Abstract

Artificial intelligence (AI) has emerged as a powerful approach for integrated analysis of the rapidly growing volume of multi-omics data, including many research and clinical tasks such as prediction of disease risk and identification of potential therapeutic targets. However, the potential for AI to facilitate the identification of factors contributing to human exceptional health and life span and their translation into novel interventions for enhancing health and life span has not yet been realized. As researchers on aging acquire large scale data both in human cohorts and model organisms, emerging opportunities exist for the application of AI approaches to untangle the complex physiologic process(es) that modulate health and life span. It is expected that efficient and novel data mining tools that could unravel molecular mechanisms and causal pathways associated with exceptional health and life span could accelerate the discovery of novel therapeutics for healthy aging. Keeping this in mind, the National Institute on Aging (NIA) convened an interdisciplinary workshop titled “Contributions of Artificial Intelligence to Research on Determinants and Modulation of Health Span and Life Span” in August 2018. The workshop involved experts in the fields of aging, comparative biology, cardiology, cancer, and computational science/AI who brainstormed ideas on how AI can be leveraged for the analyses of large-scale data sets from human epidemiological studies and animal/model organisms to close the current knowledge gaps in processes that drive exceptional life and health span. This report summarizes the discussions and recommendations from the workshop on future application of AI approaches to advance our understanding of human health and life span.

## Introduction

Aging is often described as the outcome of interactions among genetic, environmental and lifestyle factors with wide variation in life and health span between and within species (Newman and Murabito, 2013; Partridge et al., 2018; Singh et al., 2019). Exceptional life and health span represents an extreme phenotype characterized by exceptional survival (well-beyond average life expectancy), delayed onset of age-related diseases (before 80 years of age) (Pignolo, 2019) and/or preservation of good health/function relative to their peers (Perls et al., 2000, 2002; Kaeberlein, 2018). The identification of SNP associations with exceptional life and health span is a starting point for identifying targets for interventions that could potentially promote healthy human aging. In this context, understanding the functional effects of human genetic variants and cellular factors associated with exceptional survival can identify potential targets for interventions to mimic their favorable effects (Kennedy, 2008; Kaeberlein and Kennedy, 2009). The advent of high-throughput technologies is enabling the acquisition of large data sets on genetics, genomics and many cell variables, including proteins, lipids and metabolites in an effort to (1) untangle the intricate biological process of aging, (2) identify drug targets, and (3) develop therapeutics to enhance life and health span. Individually these datasets have contributed to our understanding of human physiology and diseases but existing approaches fall short in terms of understanding the complex role of those mechanisms in aging that protect individuals from age-related diseases and enable health and life span (Sebastiani et al., 2013, 2017a; Milman and Barzilai, 2015). The application of GWAS, Mendelian randomization and other similar approaches have been of limited success and have not resulted in any major breakthroughs in therapeutics for prevention of age-related diseases, including Alzheimer's Disease (AD).

Alternative strategies which could be more fruitful include integrated analysis of multi-omics data and comparative biology approaches. For example, exceptional life and health span involve a multiplicity of interacting contributory factors and the nature of these interactions can change with time. There is a need to unlock the fundamental functional information on (1) genotype—phenotype relationships, (2) causality and mechanistic action of the gene(s) at the physiologic level, (3) pleiotropic and epistatic effects that mask the influence of the gene variants, and (4) structural variations associated with these protective gene variants. Integrative multi-omics approaches hold great promise in the translation of protective variants to prevent/delay age- related diseases and promote healthy aging (Huang et al., 2017). Additionally, comparative biology of aging approaches which further investigate the factors modulating life span of long- and short-lived species could provide valuable insight into potential pathways influencing healthy aging in humans. Using data from human and animal/model organisms truly represents an opportunity that could be exploited a lot more than it has been in the past to understand the varied trajectories of aging in different species (Yanai et al., 2017).

In this context, modern data analysis and in particular, AI approaches could be transformative toward identifying strategies for preservation of good health with advancing age. AI researchers exploit computationally intense algorithms to assist humans in making sense of large, complex data sets with patterns that may not be detected using parametric statistical methods. An overarching question now in the aging field is whether AI, machine learning (ML), or deep learning (DL) approaches would be useful tools for identifying the genetic basis of exceptional health and life span. Future applications of AI in aging research appear to be diverse and may potentially contribute to the development of pharmaceutical drugs to enhance health and life span.

With this as background, The National Institute on Aging (NIA) convened a workshop in August titled “Contributions of Artificial Intelligence to Research on Determinants and Modulation of Health Span and Life Span.” The workshop invited experts in the fields of aging, comparative biology, cardiology, cancer, computer science/artificial intelligence, and bioinformatics who brainstormed ideas on how AI can be leveraged to extract knowledge from large scale data sets collected from human epidemiological studies and animal/model organisms to accomplish the following goals:

- Model the relationships between DNA, RNA, proteins, metabolites and other cell variables, associated with disease risks and exceptional healthy aging
- Infer biological models that relate genetic sequence to cellular processes
- Utilize comparative biology approaches for identifying factors which contribute to slow rates of aging
- Identify novel predictive biomarkers of aging, drug targets and gene altering therapeutics to prevent or delay age-related diseases
- Identify potential targets for pharmacological interventions to prevent age-related diseases and promote healthy aging

The overall purpose of this workshop was to assemble experts to obtain their input on ways that AI methods could be productively applied to advance our understanding of the determinants of human health and life span. Though its underlying premise was that such understanding could contribute to interventions that extend human health span and life span, it also included a focus on potential contributions of AI to understanding the basis of the wide variability of life span across species, which could further inform the development of human interventions.

### Study of Exceptional Life and Health Span—*Challenges, Goals and Resources*

Aging is the progressive accumulation of cellular changes with time that are associated with or responsible for susceptibility to various diseases and is the main risk factor for diseases. The process of aging displays wide variability across populations with some reaching extreme old- age associated with exceptionally healthy aging phenotypes (Macarron and Hertzberg, 2011). The fact that human lifespan and achievement of extreme old age, is moderately heritable motivated investigators to search for protective genetic variants/factors associated with these traits. Related to these efforts, **Dr. Daniel S. Evans** from **California Pacific Medical Center (CPMC)** in his presentation described his current strategies and challenges in data analyses that are aimed at identifying and translating protective genetic and molecular factors that drive the exceptional life and health span phenotype. The identification of SNP associations with human longevity is a starting point for identifying targets of interventions that could potentially promote healthy human aging. In the past, genome wide association studies (GWAS) of exceptional life span /longevity have identified quite a few longevity-associated variants (LAVs) (Perls et al., 2000; Willcox et al., 2006; Conneely et al., 2012; Sebastiani et al., 2013), with the most significant and widely replicated LAVs residing in the ApoE gene region. The most consistent finding is associations with the SNP marker for the ApoE e4 haplotype being associated with decreased odds of longevity (Deelen et al., 2011; Nebel et al., 2011; Broer et al., 2015). The meta-analysis of the CHARGE discovery results [(Broer et al., 2015) with published results for the FOXO3 variant rs2802292 (Willcox et al., 2006; Flachsbart et al., 2013)] revealed that the G allele was associated with increased odds of longevity at the genome-wide significance level (OR = 1.17, *P* = 1.9 × 10^−10^). A recent GWAS of Chinese centenarians identified SNPs near the genes IL6 and ANKRD20A9P (Zeng et al., 2016). A GWAS meta-analysis of centenarians of European descent replicated SNP associations at the ApoE locus and identified rare variants near the genes USP42 and TMTC2 (Sebastiani et al., 2017b). Recent GWAS of parental lifespan from the UK Biobank study have expanded the number of published LAVs (Pilling et al., 2017).

However, there appear to be a number of challenges to advance SNP associations to biological knowledge that can be used in drug development for exceptional life and health span. First, genes underlying SNP associations must be identified. The vast majority of genetic variants identified in GWAS are non-coding (Nicolae et al., 2010), which presents a challenge in identifying the gene whose function might be impacted by trait-associated variants. Second, the relevant tissue must be identified to develop an understanding of a biological mechanism and to eventually consider interventions that can target the relevant tissue. This is particularly complex since it is highly likely that multiple processes and tissues contribute to human longevity. Third, convincing evidence must be obtained that a target is causally associated with exceptional life and health span. If a target is only associated with longevity due to a confounding influence, intervening on that target will have little chance of modulating the outcome(s) of interest. Fourth, intermediate phenotypes related to a SNP's association with longevity should be identified to enable rapid testing of biological mechanisms.

GWAS, in general, operates under the common variant common disease framework, in which common variants are thought to tag haplotypes that harbor causal variants associated with common disease. Exceptional life and health span are not common conditions, and as such, might not be under the control of individual common variants. Rare variants might play a role, but it is also possible that groups of common variants and non-genetic factors could act together in high-order interactions to result in human longevity. Such high-order interactions might be rare, and standard univariate analysis performed in GWAS cannot capture complex interactions. In addition, the lack of biological knowledge of the variants is another major bottleneck in translational strategy to promote longevity. The majority of identified genetic variants identified in GWAS are non-coding, which presents a challenge in identifying the gene whose function might be impacted by trait-associated variants. This requires analyzing mechanistic pathways that underlie the aging which in turn requires omics data and functional-read outs or biological markers. Dr. Evans presented his current approach of integrating multiple lines of evidence, including eQTL studies, positional overlap, and chromatin interaction studies to link LAVs to candidate longevity associated genes (LAGs).

Mendelian Randomization (MR) analysis is also being applied to evaluate the potential impact of modulating tissue-specific LAG expression on subclinical risk and disease processes related to human longevity. Dr. Evans indicated that it still remains a challenge to identify potential intervention targets that can form the basis of translational strategies based on findings from GWAS and MR approaches. It is believed that integrated analysis of genetic and multi-omics data using AI could potentially overcome some of the hurdles that we currently have with the existing analytical strategies. AI methods with the ability to accommodate high order interactions in large datasets, might end up being successful approaches in the prediction of human longevity and the identification of factors that contribute to human longevity.

**Figure 1:**
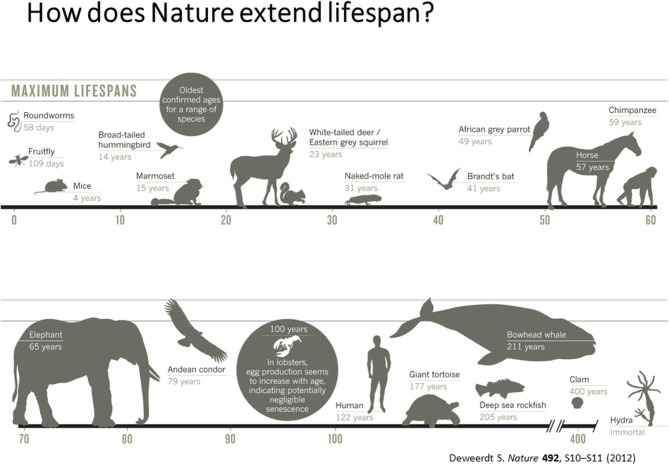
Maximum Life Span across different species [adapted from Deweerdt (2012), Copyright received from Nature Journal].

According to **Dr. Vadim Gladyshev** from **Harvard University**, the current challenges in the aging field is to understand aging as a systemic process (Gladyshev and Gladyshev, 2016; Petkovich et al., 2017) absence of biomarkers of aging and lack of knowledge on short- and long-lived states of diverse species. Hence, development of sophisticated measures of biological age by integrating various measures of biological processes is critical to develop therapeutics for health and life span. He also emphasized the need for integrating and interpreting processes that dictate short and long life in diverse species that have evolved in nature as shown in Figure 1.

Additionally, Dr. Gladyshev presented the crowd-funded and crowd-sourced system for the development of AI-powered photographic aging clocks in mice called MouseAge.org (http://www.mouseage.org).

**Dr. Evan Hadley** from the **National Institute on Aging** described resources that could be useful for developing and applying AI methods to identify factors modulating health span and life span. NIA supports a variety of human studies including family, centenarian, and longitudinal cohort studies, as well as interdisciplinary studies on mechanisms contributing to longevity and translational omics projects to identify potential therapeutic targets for interventions to extend healthy life (Table 1). NIA also supports laboratory animal projects of interventions to extend longevity and develop new animal aging models. Dr. Hadley also noted the value of comparisons among species with differing life spans to complement studies within humans, particularly through the potentially increased signal strength due to the much greater variability in life spans across species compared to their variability within humans. For example, while only a minuscule percentage of men and women survive to 1.4 times their median life spans (Bell and Miller, 2005), primate species longevities vary several folds (Table 2). These differences, particularly among such phylogenetically closely related species, may allow identification of mechanisms contributing to longevity that might not be readily detectable by human studies alone, but which could serve as the basis for developing novel human interventions. Dr. Hadley further illustrated this idea by noting that differences between rates of cancer development in humans and dogs correspond closely to differences in their life spans (Schneider, 1970), and that understanding the basis for slower rates of disease onset in humans compared to many other species might contribute to novel disease prevention strategies.

**Table 1:** Pertinent Projects.

**Project**	**Description**
Long Life Family Study	550 families with exceptional familial longevity, and spousal controls (https://longlifefamilystudy.wustl.edu/LLFS/Home.html)
New England Centenarian Study	A study of ~4,000 centenarians, siblings and their offspring since 1994 (http://www.bumc.bu.edu/centenarian/)
Longevity Genes Project	~700 exceptionally long-life span Ashkenazi Jews (https://www.einstein.yu.edu/centers/aging/longevity-genes-project/)
Kuakini Hawaii Health Span Study	~ 8,006 Japanese- American men in Hawaii as part of the Kuakini Honolulu Heart Program (https://www.kuakini.org/wps/portal/kuakini-research/research-home/kuakini-research-programs/Kuakini-Healthspan-Study)
CHARGE	Studies in the Cohorts for Heart and Aging Research in Genomic Epidemiology (CHARGE) Consortium (http://www.chargeconsortium.com/)
Longevity Consortium	Centenarian studies, comparative biology of longevity, pilot mechanistic studies (https://www.longevityconsortium.org/)
Longevity Genomics Network	Planning and informatics resource development for translating human genetic findings (https://www.longevitygenomics.org/)
Animal Intervention Testing Program	Testing intervention effects on mouse longevity and health span (https://www.nia.nih.gov/research/dab/interventions-testing-program-itp)
Animal Models Program	Development of new models for aging research, comparative biology of aging (https://www.nia.nih.gov/research/dab/interventions-testing-program-itp)

**Table 2:** Plasticity of life span in primates.

**Species**	**Maximum reported life span (years)**
White-tufted-ear Marmoset	23
South American Squirrel Monkey	30
Rhesus Monkey	40
Brown Capuchin	46
Chimpanzee	59
Human	122

*Source: Animal Ageing and Longevity Database http://genomics.senescence.info/species*.

**Figure 2:**
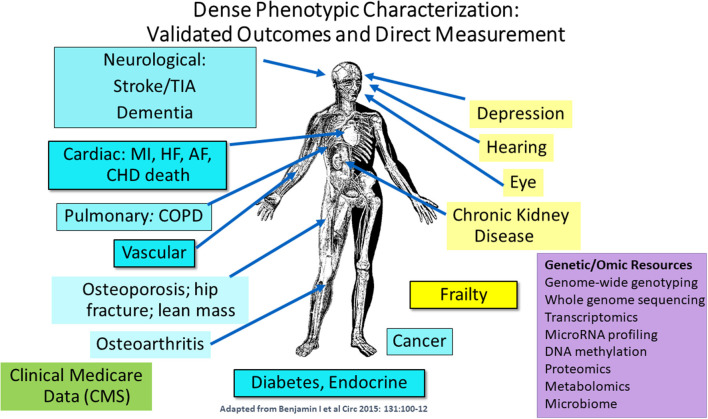
Extensive phenotypic characterization of the cohorts. CMS indicates Centers for Medicare and Medicaid Services data [adapted from Benjamin et al. (2015). Copyright received from Circulation Journal].

**Dr. Joanne Murabito** from **Boston University** elaborated more on human cohort studies that have acquired data on many thousands of people of including some of quite advanced age as enabled by technological advances. Most importantly, longitudinal epidemiologic cohort studies have deeply characterized participants often over the adult lifespan with respect to age-related chronic conditions, physical function and cognition with repeated in-person examinations and validation of outcomes. The goal of this characterization is to identify common and low frequency genetic variants and causal factors associated with exceptional longevity. She presented a descriptive table (Table 3) listing a collection of a sample of cohorts with the available data for analyses. Cohorts have extensive biorepositories (Figure 2) for testing an array of non-genetic biomarkers and creating extensive genetic/genomic resources. Health behaviors, risk factors, and imaging (ECG, echocardiography, carotid ultrasound, coronary calcification, brain MRI, bone mineral density) to characterize subclinical measures of disease are available.

**Table 3:** Human cohort studies (Partial List).

**Cohorts**	**# of Subjects**
Atherosclerosis Risk in Communities (ARIC)	15,368
Coronary Artery Risk Development in Young Adults (CARDIA)	5,114
Cardiovascular Health Study (CHS)	5,888
Framingham Heart Study	≈15,000
Hispanic Community Health Study/Study of Latinos (HCHS/SOL)	16,415
Jackson Heart Study (JHS)	5301
Multi-Ethnic Study of Atherosclerosis (MESA)	7,071
Health Aging and Body Composition (Health ABC) Study	3,075
Study of Osteoporotic Fractures (SOF)	9,704
Osteoporotic fractures in men (MROS)	5,994
Women's Health Initiative (WHI)	161,808
Age, Gene/Environment Susceptibility study (AGES-Reykjavik)	5,764
Baltimore Longitudinal Study of Aging (BLSA)	>3,200
Invecchiare in Chianti (InCHIANTI)	1,453
Long Life Family Study (LLFS)	4,953
SardiNIA	6,700
Memory and Aging Project	1,200
Whitehall II	10,308

*Dawber et al. (1951); Feinleib et al. (1975); Friedman et al. (1988); Atherosclerosis Risk in Communities (1989); Fried et al. (1991); Cummings et al. (1995); Women's Health Initiative (1998); Bild et al. (2002); Blank et al. (2005); Orwoll et al. (2005); Taylor et al. (2005); Wilson et al. (2005); Harris et al. (2007); Splansky et al. (2007); Sebastiani et al. (2009); Newman et al. (2011); Conomos et al. (2016)*.

Investigators are now trying to overcome challenges to combine data across cohorts including study design (case/control, cohort, family-based, biobank), phenotype definition, frequency of measurements, and missing data. There are a number of large and productive consortia (CHARGE, TOPMed, Longevity Consortium (LC), Cross-Cohort-Collaboration) that include cohort investigators addressing some of these challenges. Harmonization for some age-related phenotypes is underway using principles from the Maelstrom Research Guidelines for Rigorous Retrospective Data Harmonization (Fortier et al., 2017). Leveraging the clinical, biomarker, imaging and genetic/omic data across cohorts in future studies is believed to increase our understanding of healthy aging in multiple systems.

**Dr. Richard A. Miller** from the **University of Michigan** mentioned the availability of datasets from NIA supported non-human studies (multiple species including primates and mammals) and an animal intervention testing program (ITP) with interventions that increase mouse longevity and health span. He provided examples of few compounds such as Rapamycin, 17Alpha estradiol and Acarbose that extend lifespan in mice. Details on the work conducted by the ITP can be obtained from the link (https://www.nia.nih.gov/research/dab/interventions-testing-program-itp). He described the ITP work based on a genetically heterogeneous four-way cross study providing opportunities for mapping polymorphic alleles and a greater degree of robustness than for work in a single inbred mouse stock, such as C57BL/6. Resources from the ITP studies include (a) data on lifespan, weight, and terminal pathology for many of these mice, (b) DNA for over 8000 mice; (c) fixed specimens for nearly all mice; (d) tissue blocks and slides for a series of cross-sectional and end-of-life necropsy studies. NIA has also been supporting a program that develops animal models, both for vertebrate and invertebrates for comparative biology studies in aging.

### The Promises of AI—Can AI Revolutionize Aging Research?

AI is expected to revolutionize many aspects of society through its ability to optimize processes and decisions using computer-based algorithms. Given the current challenges in data mining, the aging field is exploring supplemental and alternative approaches such as AI in the modern aging research agenda to identify the underlying cellular biology of aging processes and to determine the mechanisms of exceptional aging processes. The aging field is a bit different than many other areas of biomedicine because and do not yet have good measures of biological age. Thus, AI is needed to define basic measures of aging whereas many other disciplines can more directly measure their outcomes of interest. There are several other areas where AI has the potential to have a positive impact. Cohort studies on aging offer extensive longitudinal phenotype data and an array of genetic and omic data to study exceptional life and health span. The Monarch Initiative (https://monarchinitiative.org), an international consortium focused on integrating disease, gene, and phenotype data from many species has created a rich resource of data from different species. The datasets in Monarch can be used to integrate and make connections among other biological entities of interest, such as genes, genotypes, gene variants, models (including cell lines, animal strains, species, breeds, as well as targeted mutants) biological pathways, human orthologs and phenotypes. By leveraging such big data from humans and model species to allow computers to learn, AI is expected to vastly improve recognition of patterns and relationships, allowing for broad applications in complex biological processes associated with aging.

Under the umbrella of AI, ML has succeeded in complex tasks by trading experts and programmers for data and non-parametric statistical models. However, the applications for which ML has been successfully deployed remain limited, especially in aging. We expect this change in the near future for several reasons. First, AI can complement statistical approaches to data analysis by making fewer assumptions about the model, improving the power to detect non-linear interactions, automating model discovery, and through incorporation of biological and clinical knowledge from domain experts or from prior research results. Second, AI is better powered for prediction and forecasting than parametric statistical approaches that are designed for inference. Third, some AI methods such as Bayesian belief networks can infer causality that will be important to provide insight into the etiology of aging and aging-related diseases. This is important because many AI methods consist of models based on associations that don't necessarily reflect causal factors. Fortunately, AI methods can be applied to any study design that parametric statistical approaches can be applied to. Of course, issues such as bias and confounding that plague any study design and data analysis approach are of concern with AI approaches and must be taken into consideration. Larger sample size may be required if the goal of the AI approach is to detect non-linear interaction among the features being studied. Additional challenges lie in the interpretation of AI models and results that are often more complicated and less transparent than those derived from parametric statistical models. This will be important address as the aging community increasingly embraces AI approaches.

There are several reasons why now is the time to adopt AI approaches. First, high-performance computing is inexpensive and accessible. The widespread construction of local computing infrastructure and the availability of cloud computing make the implementation of AI approaches more tractable. Second, AI has matured rapidly as a field and is starting to have real successes in a variety of domains of biomedical research. An example is DL neural networks that have had success in areas such as image analysis. Third, we have extensive knowledgebases such as PubMed that provide the research memory or the training data that AI methods need. Fourth, as we review later, significant investments in aging and longevity research over many decades have provided a wealth of data that need to be integrated and analyzed using both statistical and AI approaches. Finally, AI is increasingly accessible as automated methods and user-friendly software emerge and an emphasis is placed on interpretation. We recognize that AI is in its infancy and there are still a number of challenges that must be overcome before AI can have a major impact in aging research. However, now is the time to address these challenges and to begin adding this technology to the toolkit used by AI researchers. An important early challenge is to identify those aging problems and research questions that can benefit from an AI approach. A starting point is to identify a list of published aging studies where parametric statistical approaches were used and for which the data are publicly available. Reanalysis with AI methods along with a thoughtful comparison to original results would provide a good foundation for planning future studies using AI.

A brief introduction to AI as summarized by **Drs. Srinivas Kankanahalli** from **ClearAvenue, David Jacobs** from **University of Maryland and Jason H. Moore** from **University of Pennsylvania** is provided below to educate researchers studying aging. A review of the opportunities for AI researchers to have an impact in this domain along with a set of recommendations that resulted from the NIA workshop on AI and aging is summarized below.

### History of AI

AI arose in the 1940s and 1950s in parallel with the development and use of the first mainframe computers (Russell and Norvig, 2003). The phrase AI was coined during a workshop at Dartmouth College in 1956 to unify the various early developers of the discipline. There was a lot of excitement at that time about the potential of AI for solving difficult problems. This excitement grew as computers became faster and more accessible through the 1960s and 1970s. Despite the excitement, it became apparent that AI was not living up to the hype. This led to what has been called the AI winter, a period through the late 1980s and 1990s when investment in AI research and commercial efforts slowed significantly. In recent years, excitement about AI has increased due to some high-profile successes. One example of this is the successful launch of Watson AI by IBM and its much-publicized defeat of the best human Jeopardy contestants in 2010 on live television (Ferrucci, 2010). Part of this current success of AI can be attributed to availability of the computational resources that are needed for these algorithms to solve hard problems.

**Figure 3:**
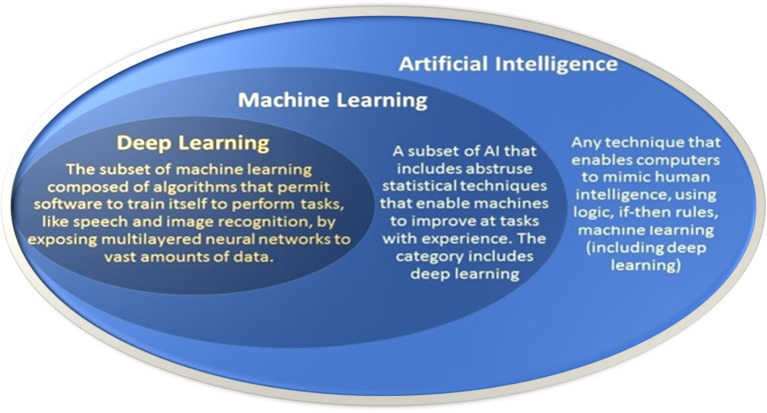
Relationship of AI, ML, and DL (Modified from Toward Data Science).

Essentially, AI refers to computer software that can reason and adapt based on sets of rules and data. The original goals for AI were to mimic human intelligence. Early AI systems relied heavily on expert-derived rules for replicating how people would approach these tasks. ML, a subfield of AI, emerged as research began to leverage numerical techniques integrating principles from computing, optimization, and statistics to automatically “learn” programs for performing these tasks by processing data: hence the recent interest in “big data.” ML is a field of AI and is based on computational statistical algorithms that allow computers to learn directly from data, without being explicitly programmed to perform a specific task. Often, ML uses *training* data to fit a parametric model that can predict important features of new *test* data. Thus, for example, ML techniques have the potential to automatically identify the most important features related to key differences in patient data, that is, disease vs. healthy. The potential applications of ML in healthcare are vast, including screening, disease detection and classification, patient risk stratification, and optimal therapy selection (Topol, 2019). DL is a specific ML approach that employs multilayered neural networks (Goodfellow et al., 2016). These networks are composed of layers of computational units, dubbed “neurons,” that apply a non-linear function to the weighted sums of their inputs. These units are loosely inspired by the behavior of biological neurons. DL is distinguished in part by its ability to make use of very large training sets. Figure 3 illustrates the relationship of AI, ML, and DL.

### Definition and Components of AI

AI has been defined in many different ways, but the common thread refers to intelligent decision-making by computers. Intelligence could refer to biological decisions made by simple organisms such as bacteria during chemotaxis or much more complex decisions made by humans. For the purposes of our discussion here we will focus on AI as an approach to data analysis that can identify patterns as the human brain does and that makes analytical decisions (e.g., what method to use) as a human analyst would using their expert knowledge and training. There are several important components that are useful for building a computational framework that can analyze data as a human expert. The first is knowledge representation that attempts to put what is known about a problem in a format that is optimal for the computer to work with. For the Jeopardy problem this meant representing the knowledge in Wikipedia and other sources such that Watson could efficiently make use of it. A related area is knowledge engineering that is used to transfer the knowledge that a human expert has into a computer representation (Ferrucci, 2010). An example is the rules that clinicians use to make patient treatment decisions. A second major component of AI is ML that allows a computer to learn from experience. The most common context for ML is data analysis where a model of the data is constructed and then evaluated. Feedback from the quality of the model is then used to build a new model. The computer learns over time what types of models are successful. A third major component is reasoning. Reasoning can be deductive or inductive with the goal of making a decision (e.g., which treatment to give the patient). The combination of a knowledge representation and engineering ML, and reasoning can be used to build a computational system that attempts to approximate the problem-solving ability of the human brain. There are several other components of AI such as natural language processing that are important depending on the application.

### MYCIN as Early Example of AI in Medicine

Artificial intelligence has had a long history in medicine. One of the early successes was the MYCIN system for prescribing antibiotics to intensive care unit patients (Shortliffe et al., 1975) that was based on a type of AI called an expert system (Duda and Shortliffe, 1983) The MYCIN system included a knowledgebase of rules from clinicians (i.e., knowledge engineering), a database of facts about patients (i.e., the data), and an inference engine that would produce treatment decisions using probability (i.e., reasoning). It was demonstrated that MYCIN made antibiotic treatment decision as well or better than human experts (Yu et al., 1979). However, it was never used in clinical practice because of legal concerns and the data required took 30 min to enter into the computer. Despite never being used, MYCIN demonstrated the potential of AI in medicine.

### IBM Watson as Modern Example of AI

As mentioned above, IBM Watson has served as a modern example of the potential for AI in medicine. Building on the success of Watson for playing Jeopardy, Watson was adapted for use in the healthcare domain with an initial focus on oncology. Watson was adopted by several academic medical centers as a commercial product for improving decision making. Watson has also proved useful in other domains. For example, Watson for Genomics was used by a molecular tumor board to identify actionable mutations from the literature that were not accounted for in their current lists (Rhrissorrakrai et al., 2016; Wrzeszczynski et al., 2017; Patel et al., 2018). This is a useful application of AI because human experts have limited time to study the literature for new clinically useful findings. Currently, **Dr. Laxmi Parida**, a senior researcher in **IBM**, is exploring AI/M approaches to analyze the Alzheimer's Disease (AD) WGS data from ADNI and ADSP- CC (~3,500 samples). She discussed the usefulness of Topological Data Analysis (TDA) as a promising new mathematical approach on complex discrete datasets such as aging.

### What Is Deep Learning?

Neural networks have a long history in AI and have been widely used as a ML method for identifying non-linear patterns in data. Their development was inspired by neurons in a brain whose connections can be strengthened through positive reinforcement. They have regained popularity with the availability of high-performance computing that has allowed deep neural networks with many nodes (i.e., neurons) and connections (Krizhevsky et al., 2012; Lecun et al., 2015). These deeper and more complex neural networks have made it possible to accurately classify images. Applications include the identification of melanoma from photos of skin lesions (Haenssle et al., 2018) and risk stratification from radiographic images of lung cancer (Hosny et al., 2018). Few investigators have provided in-depth reviews of DL methods and applications in biology and medicine (Topol, 2016; Ching et al., 2018). Although DL has had a number of important successes there are some limitations. The most important limitation relevant to aging researchers is the greatly increased difficulty in interpretation of the resulting models due to the size and complexity of the networks. This can be problematic if the goal is to understand why the model works so that new experiments can be planned. Further, many of these models have not been validated in the clinic or in prospective studies. There is no doubt that DL has an important place in the AI universe and will enable new biomedical applications.

### Automated ML/AI for Data Analytics

One of the most important challenges in using ML to detect complex patterns in big data is the selection of the algorithm to be used. There are many different ML methods, and each looks at the data in a different way using different mathematical models and computational approaches. It is difficult to know which ML method is the right one for your data. This was highlighted in a recent paper by Olson and La Cava (Olson et al., 2018) that compares the performance of a large number of different ML methods across 160 publicly available data sets of different sizes and shapes (Olson et al., 2017). This paper showed that although there were methods such as gradient boosting that performed well on a majority of data sets there was not one method that performed best across all data. In fact, every method performed best on at least a small number of data sets. Selecting the right method is particularly challenging for non-experts. To address this issue there have been several methods developed specifically to automate the selection of ML methods and their parameter settings. These include Auto-Weka (Thornton et al., 2013) built using the Weka ML software, AutoSklearn using the scikit-learning library (Feurer et al., 2015), Penn AI (La Cava et al., 2019) and the Tree Based Pipeline Optimization Tool or TPOT (Olson, 2016) that builds an entire ML pipeline using all the components of scikit-learn as building blocks. These methods and their software are all freely available and open-source. The TPOT approach is specifically designed for biomedical problems and includes operators for dealing with big data (Sohn et al., 2017). These methods represent an important step toward true AI for tackling big data problems in biomedical research. The value for aging researchers is that automated approaches take the guesswork out of which methods and parameters settings to pick thus making this technology more accessible to a wide audience.

### A Few Examples on Application of AI Approaches in Bio-medicine

Having observed the entry of AI in biomedical fields in the recent past for disease diagnosis and drug development, few experts were invited to narrate their experiences on the challenges and successes of AI approaches in their respective area of research.

**Dr. Rahul Deo**, a cardiologist at **Brigham Young hospital**, in an effort to overcome challenges associated with early disease detection, exploited automated approaches in a pilot study to resolve cardiac disease heterogeneity. He hypothesized that failure to recognize cardiac heterogeneity is hindering the discovery and validation of novel treatments for heart diseases. By employing novel ML approaches such as automated interpretation of echocardiography and electrocardiography, and integration of clinical, imaging and molecular data to define disease subtypes, he was able to track dysfunction reflecting the underlying disease states.

**Dr. Rick Stevens**, from **Argonne National Labs**, is leading the NCI-DOE collaboration on the Joint Design of Advanced Computing Solutions for Cancer and working on applying large-scale computation to the problem of predictive oncology. He presented interesting ML models for predicting drug response in Cancer. The team has developed ML models for both single drug and drug combinations that are trained on drug response datasets from screens conducted on cell lines and patient derived xenografts.

He also mentioned major challenges in predictive oncology that include integrating data between studies for largescale model training, developing effective representation for drugs and devising model formulations that generalize well across cancer types and drugs. DL models get their predictive power from, often inscrutable, extremely high dimensional models. An estimate of the total uncertainty, including both statistical fluctuations and modeling limitations, in this setting needs development of new techniques beyond what are used in standard statistical analysis used for less complex models fitted without access to large data sets.

**Dr. Steven R. Cummings** from **CPMC** described projects underway, in collaboration with Google Accelerated Sciences, on the application of DL to images of the human body and cells to improve prediction of aging outcomes. However, a few issues have arisen in developing projects to apply AI to existing human cohort studies. First, procuring the necessary sample sizes has been challenging. The number of images available from human cohorts with subsequent aging outcomes are limited. Thus, it is important to determine early in the process whether it will be feasible to assemble the required amount of data. Second, it has been difficult to determine how many individuals with images or samples of tissues or cells are needed to develop validated and generalizable predictions. The answer from practitioners of AI to “how many?” is generally “more.” In standard sample size estimation, fewer individuals or samples are needed for “prediction” of continuous endpoints, such as change in walking speed with aging, then for incident outcomes, such as mobility and disability. Ongoing projects are using data with thousands of images and hundreds of outcomes, taking advantage of strategies to initially train a neural network on smaller samples. Importantly, there are often barriers to collaborations between Industry, Institute sponsors of NIH-funded cohorts, and their academic institutions.

Many academic institutions and some NIH institutes (not NIA) have reticence to share data with commercial groups, and several issues, such as intellectual property, HIPAA, material transfer agreements, and funding can substantially delay projects. There is a need for model agreements between NIH institutes and commercial entities that can serve as models for these collaborations. **Phil Nelson and Dr. Kai Kohlhoff** from **Google** showcased a few projects (unpublished data) where teams have been working on the application of DL approaches to achieve expert-level accuracy in determining diabetic retinopathy, unsupervised clustering of drugs by dose and mechanisms of action in cell images, and automatic staining of cell components. With respect to aging research, the Google team has used the Alzheimer's Disease Neuroimaging Initiative (ADNI) dataset consisting of 13,000 MRI images on 1,500 subjects and transfer learning to infer AD diagnosis and disease progression with a network pre-trained on the ImageNet dataset (unpublished).

**Dr. Alex Zhavoronkov** from **Insilico Medicine** provided an overview of the recent advances in the applications of DL to the development of aging biomarkers, target identification, generation of synthetic human data using the generative adversarial networks (GANs) using age as a generation condition (Zhavoronkov et al., 2019). He demonstrated the application of the deep neural networks for the prediction of chronological age of patients using the basic anonymized clinical test data available for public testing using the aging.ai system. The hematological aging clocks (Putin et al., 2016) were tested in multiple populations to explore population-specificity and establish biological relevance (Mamoshina et al., 2018a) and evaluate the effects of lifestyle and behavior (Mamoshina et al., 2019). Insilico medicine has also achieved integration of multi- modal data for aging research by launching the intelligently-formulated nutraceuticals (Aliper et al., 2016) and establishing a real-world data collection effort with the launch of the young.ai system. Dr. Zhavoronkov provided examples of how deep neural network approach has been applied on the blood biochemistry, transcriptomic (Mamoshina et al., 2018b) and imaging data (Aliper et al., 2016) as well as the other data types to predict chronological and biological age of individuals. He also showed preliminary data with successful application of AI on deep feature selection algorithms for target identification and biomarker development. To rapidly validate the targets in biological assays Insilico Medicine developed a set of GAN and reinforcement learning (RL) systems to generate novel molecules with the desired set or properties (Putin et al., 2016; Kadurin et al., 2017; Polykovskiy et al., 2018). Insilico Medicine scientists contributed to the development of a large public database of biomedical funding and literature related to aging called AgingPortfolio.org (Zhavoronkov and Cantor, 2011; West et al., 2018) and open database of geroprotective drugs and lifespan experiments that can be used for training: DrugAge (Moskalev et al., 2015; Barardo et al., 2017; http://genomics.senescence.info/drugs/) and Geroprotectors.org (https://geroprotectors.org/). It also contributes to the development and curation of the open community database of pathways implicated in aging and longevity, AgingChart.org (Moskalev et al., 2016; https://agingchart.org/).

**Calico**, represented by **Dr. Jun Xu** is applying AI approaches to understand fundamental biology that controls life span in model organisms. Calico has built an end-to-end deeplearning pipeline to automate the analysis of yeast replicative lifespan data collected from custom microfluidic devices (unpublished). Deep convolutional neural network models are being used to predict cell-type-specific epigenetic and transcriptional profiles in large mammalian genomes, based on DNA sequence alone with the hope of unraveling cellular processes that dictate life and health span.

**Dr. Atul J. Butte**, from **University of California, San Francisco** from his studies applying AI approaches demonstrated the conversion of trillions of points of molecular, clinical, and epidemiological data measured by researchers and clinicians over the past decade into diagnostics, therapeutics, and new insights into disease. He mentioned the promise of DL approaches in evaluating a patient's diagnosis, prognosis from the eHR and ICD codes with a decent amount of accuracy (Rajkomar et al., 2018). He elaborated on the process within the University of California wherein the six health systems collect and store eHR in the common open standard Observational Medical Outcomes Partnership (OMOP) Common Data Model (CDM). In one specific example, he showed how deidentified data for patients at one UC medical center was connected to events recorded in data containers based on the Fast Healthcare Interoperability Resource (FHIR) specification. The FHIR resources are placed in temporal folders depicting all events recorded in eHR (timeline). The DL process then uses this historical data to make predictions for each patient. He showcased few proof of principle studies that used data combining healthcare data from across the six UC medical schools and systems.

**Dr. James Cole** from **King's College, London** presented his work on a potential brain-aging biomarker, so-called 'brain age', derived using ML analysis of structural neuroimaging data. He showed interesting data applying the identified biomarker from his ML analysis to study brain aging in general population and patients with diseases such as Down's syndrome, HIV, traumatic brain injury, epilepsy, multiple sclerosis and Alzheimer's. Dr. Cole also outlined how this brain aging biomarker has shown prognostic value, predicting survival time in a large of older adults in Scotland, and how predictions of brain age can be combined with the epigenetic clock in order to refine these mortality estimations (Cole, 2018).

**Dr. Paola Sebastiani** from **Boston University** presented her analysis using Bayesian model—based clustering of longitudinal trajectories of neuropsychological scores to identify distinct patterns of cognitive decline in the domains of episodic memory, attention, processing speed, and verbal fluency. The data were collected in participants from the Long-Life Family Study (LLFS), a unique cohort of families with clustering of exceptional survival. The analysis discovered predictors of multi-domain cognitive change such as gait speed, and domain-specific predictors of cognitive change such as low IL6 and NTproBNP, or the APOE2 genotype that predict slower change of processing speed. The correlation between patterns of changes of cognitive functions in multiple domains and patterns of changes of physical functions corroborated other reports that showed aging affecting many domains simultaneously which is the basis for “a common cause theory of aging.”

**Dr. Haiyuan Yu** from **Cornell University** showed an ensemble-classifier approach “ECLAIR” for proteome-scale 3D interactome modeling and its applications in precision medicine. While simply knowing which proteins interact with each other provides valuable information to spur functional studies, far more specific hypotheses can be tested if the spatial contacts of interacting proteins are known. Using ECLAIR, he has created the first multi-scale proteome-wide structural interactome in human for all 122,647 experimentally-determined binary interactions reported in major databases.

**Dr. Sudha Seshadri** from **University of Texas Health Science Center at San Antonio** discussed the use of ML, AI and systems biology approaches in 3 specific settings: (i) genome- wide voxel-based analyses of MRI data in the CHARGE consortium, (ii) trial ready biomarker discovery in the MarkVCID consortium and (iii) whole genome, epigenome, gene expression, metabolome and proteome-based exploration of the heterogeneity and novel biology underlying dementia. The Framingham Heart Study has high dimensional genomic (GWAS and WGS), multi-omic (DNA methylation, gene expression in blood and multiple brain regions, miRNA, metabolome, proteome and microbiome) as well as comprehensive phenomic data that extends over the participant's lifespan (repeated brain MRI and cognitive function measures, amyloid and tau PET imaging, incident stroke, dementia, and information on subclinical function, biomarkers and clinical disease in various systems (cardiovascular, pulmonary, hepatic, renal, bone, joint, and inflammatory). Such high dimensional data provided an exceptional opportunity to uncover novel biology and repurposable drug targets for disease and resilience and to craft better risk prediction scores using novel computational and systems biology approaches.

### Future Opportunities for AI in Aging

The workshop focused both on AI analyses of existing data, and on the need for new primary data that would allow productive application of AI methods for increased understanding of determinants of longevity and health span. Given these focuses, the speakers were requested to provide their insights on the following questions:

- How can AI approaches be applied to existing genetic/omics and other phenotype data from aging studies?
- Is there a need for more primary data (e.g., bigger Ns, greater data density on individuals, data from various race-ethnic and geographic groups, data from more species, data integration across cell and tissue models, model organisms and humans) to allow key AI analyses?
- Is there a need for new AI computational methods to address special features of life span data (e.g., longitudinal changes, birth cohort effects, differential survival and generation of synthetic data)?
- What are the longer-term prospects for AI in aging research as AI methods develop further?
- What types of training are needed for aging researchers to utilize advances in AI tools?

### The Overall Recommendations From the Speakers Are Summarized as Below

#### Research Infrastructure

- Creation of an open data commons for aging research to enable collaborations. This could include international organizations with relevant data on aging and serve as a venue to foster collaborative studies across international cohort studies.
- Incentivize and attract computationally oriented research groups to apply AI methodologies on data from aging research.
- Reduction of barriers for appropriate access, analysis, interpretation and application of data to increase likelihood of successful discovery of biomarkers, target(s) and interventions.

#### Research Programs

- Initiation of research programs which will foster collaborations between scientists in aging research and computational scientists working on applying AI & ML to social, biological, neuroscience and medical applications.
- Guidance on sample sizes required for AI and other issues such as bias, confounding, and model interpretation would be valuable for determining the feasibility of applying AI to measurements in existing cohorts.
- Planning hackathon competitions for curation of data from aging studies and to develop novel AI algorithms for aging-relevant data mining.
- Development of pilot feasibility studies on existing data where published AI methods are applied in an initial discovery phase, followed by a Refine/Development Phase of AI methods to address data volumes challenges such as transfer learning and generative methods to reduce the needs for large-volumes of data.

#### Education and Training

- Initiation of NIH Career (K) awards to provide support training of senior postdoctoral fellows or faculty-level candidates in the United States on AI. The objective of these programs is to bring candidates to the point where they are able to conduct their research independently and are competitive for major grant support.
- Institutional training (T32) grants with a primary focus on AI (both biologists and computational scientists). These are training grants made to institutions in the United States would support groups of pre- and/or postdoctoral fellows, including trainees in basic, clinical, and behavioral research. Major purpose of this training grant is to ensure the availability of diverse and highly trained workforce to assume leadership roles in the application of AI approaches to biomedical, behavioral, and clinical research.

## Conclusions

AI approaches appear to be extremely valuable for integration of genetic and cellular data from human and other species and for modeling biological processes associated with aging. Such analyses could potentially resolve several unanswered questions currently pending in aging research. It is hoped that researchers in the aging field could collaborate, share resources and computational tools for mining the wealth of genetic and multi-omics data for novel discoveries to enhance health and life span. AI approaches could complement standard statistical approaches which are designed for inference and reveal complex patterns missed by parametric models that assume a particular functional form and could serve as a predictive tool in data analysis Aging and longevity are influenced by many interacting components and AI is particularly well-suited for modeling complex patterns driven by non-additive interactions and genetic or phenotypic heterogeneity.

## Workshop Speakers and Organizers

Evan C. Hadley, M.D., Chhanda Dutta, Ph.D., Max Guo, Ph.D., Marilyn Miller, Ph.D., Daniel S. Evans, Ph.D., Joanne Murabito, M.D., Vadim Gladyshev, Ph.D., Richard A. Miller, M.D., Ph.D., Srinivas Kankanahalli, Ph.D., David Jacobs, Ph.D., Atul J. Butte, M.D., Ph.D., Rick Stevens, Ph.D., Rahul Deo, M.D., Haiyuan Yu, Ph.D., Steven R. Cummings, M.D., Phil Nelson, Kai Kohlhoff, Ph.D., James Cole, Ph.D., Alex Zhavoronkov, Ph.D., Laxmi Parida, Ph.D., Paola Sebastiani, Ph.D., Sudha Seshadri, M.D., and Jun Xu, Ph.D.

## Author Contributions

JM and NR wrote and edited the manuscript with substantial input on contents/edits from the Workshop Speakers.

### Conflict of Interest Statement

The authors declare that the research was conducted in the absence of any commercial or financial relationships that could be construed as a potential conflict of interest.
